# Sarcoma Excision and Pattern of Complicating Sensory Neuropathy

**DOI:** 10.1155/2014/168698

**Published:** 2014-03-24

**Authors:** Neil R. Wickramasinghe, Nicholas D. Clement, Ashish Singh, Daniel E. Porter

**Affiliations:** ^1^University of Edinburgh, General Surgery Unit, Royal Infirmary of Edinburgh, 51 Little France Crescent, Edinburgh EH16 4SA, UK; ^2^Edinburgh University, 4A Howe Street, Newtown EH36TD, UK; ^3^Edinburgh Orthopaedic Trauma Unit, Royal Infirmary of Edinburgh, 51 Little France Crescent, Edinburgh EH16 4SA, UK

## Abstract

A potential complication of sarcoma excision surgery is a sensory neurological dysfunction around the surgical scar. This study utilised both objective and subjective sensation assessment modalities, to evaluate 22 patients after sarcoma surgery, for a sensory deficit. 93% had an objective sensory deficit. Light touch is less likely to be damaged than pinprick sensation, and two-point discrimination is significantly reduced around the scar. Results also show that an increased scar size leads to an increased light touch and pinprick deficit and that two-point discriminatory ability around the scar improves as time after surgery elapses. 91% had a subjective deficit, most likely tingling or pain, and numbness was most probable with lower limb sarcomas. Results also demonstrated that there were no significant relationships between any specific subjective and objective deficits. In conclusion, sensory disturbance after sarcoma surgery is common and debilitating. Efforts to minimize scar length are paramount in the prevention of sensory deficit. Sensation may also recover to an extent; thus, sensory reeducation techniques must become an integral aspect of management plans. Finally to obtain a comprehensive assessment of sensory function, both objective and subjective assessment techniques must be utilised.

## 1. Introduction

Sarcomas are rare mesenchymal malignancies originating in supportive/connective body tissues including muscle; neural, cartilaginous, vascular, and adipose tissue; and bone [1–6]. There are approximately 3200 sarcomas diagnosed in the UK each year [7]; they account for 1% of malignant neoplasms in adults and 10% in children [8]. Although sarcomas are infrequent neoplastic manifestations [9], they impact substantially on mortality (50% 5-year survival) [10, 11]. The majority (60%) of sarcomas originate peripherally, 15% affect the head/neck/external trunk, and the remainder are in the retroperitoneal abdomen [12].

In the 1970s, amputation was the cornerstone of sarcoma management. Presently, limb salvage surgery is often preferential [13]; this surgical modification along with contemporary furtherance in imaging, biomedical engineering, and the advent of adjuvant chemotherapy has greatly improved survival [12, 14, 15]. With improved survival rates, complications of sarcoma management afflict all aspects of a patient's health for longer [16]. A frequently overlooked complication of sarcoma surgery is neurological (specifically sensory) impairment. As many as 73% of patients have developed a new subjective neural impairment after tumour excision, with the majority experiencing a transient mild sensory loss, while some experienced major sensation impairment [17]. Sensory nerve injury has also been documented following other extremity surgeries, such as varicose vein surgery [18, 19] and lower limb arterial surgery [20].

Due to the profound detrimental impact that sensory dysfunction has on patients [21] and the contemporary shift in medicolegal conventions towards a litigation culture [22], it is crucial that patient and clinician are made aware of all aspects of this potential management complication.

The present study utilised objective and subjective sensory measurement tools, to specifically assess the likelihood of a sensation deficit occurring after sarcoma excision surgery, and subsequently quantified and characterized these deficits. The study also aimed to establish any apparent predisposing/precipitating factors in the occurrence of both objective and subjective sensory deficit, with a view to informing future management plans. The study also addressed the question of whether subjective sensory dysfunction correlates to objective sensory dysfunction in individual patients, an important matter with regard to the accurate assessment of a patient's neuropathy [23].

## 2. Method

### 2.1. Recruitment

Over a period of 12 months, 22 patients who had undergone sarcoma excision surgery were identified and recruited, as they attended a sarcoma follow-up clinic, at the Cancer Centre in the Western General Hospital, Edinburgh. There were 13 male patients and 9 female patients, with a mean age of 58 (16–84). There were 9 upper body tumours (defined as occurring superior to the anterior superior iliac spine (ASIS)) and 13 lower body tumours (inferior to ASIS).

### 2.2. Surgery

Sarcoma excision surgery was performed by a consultant orthopaedic surgeon; 20 patients underwent limb-salvage surgery, and 2 had above-knee amputations.

### 2.3. Questionnaire

The questionnaire formed the subjective evaluation of sensory function [24]. Having gained written consent, patients were asked to complete a questionnaire about sensation changes following surgery. The questionnaire asked about tumour location and subsequent surgical scar. Patients were then asked whether they had experienced any sensory neurological symptoms (specifically sensation loss, tingling, or numbness) before/after their surgery and whether these symptoms had spread to other parts of the body. Patients were also asked whether, excluding postoperative pain, they had ever experienced pain around their scar, and if so, they were asked to rate this on a pain scale [25, 26] and identify whether any analgesics had been taken. Finally, patients were asked whether any of their symptoms after surgery had caused them inconvenience or resulted in a reduction of limb function.

### 2.4. Tests of Sensory Nerve Function

The objective evaluation comprised three tests of sensory nerve function. Prior to conducting the tests, scar length was measured in millimeters.

The first test assessed light touch (LT). A cotton wool ball was dabbed on the patient's skin around the scar, while the patient was blindfolded, and the patient was asked to respond verbally to each touch they felt [27]. The same location on the contralateral (unoperated) side of the body was also assessed as the control.

The second test assessed superficial pain. A neurotip (neurological tool to assess pinprick (PP)) was touched on the patient's skin around the scar (at the same points the cotton wool was dabbed), while the patient was blindfolded, and the patient was asked to respond verbally to each touch they felt [27]. The contralateral side of the body was tested in the same way as in test one for use as a control.

For both tests the cotton wool/neurotip was placed 0.5 cm from the scar and at the same positions along the scar; if sensation was absent at a point, the stimulus was moved a further 0.5 cm perpendicular to the scar, and sensation was tested at this point. This was repeated until the point that sensation was present. Thus the boundaries of the areas with absent sensation could be marked on a diagrammatic representation of the patient's scar, and utilizing the measurement of scar length and the knowledge of how many points were tested along the scar's length, the LT/PP area deficit could be calculated. The third test assessed two-point discrimination. A pair of blunt-ended calipers was placed on the patient's skin at several positions around the area of the scar, while the patient was blindfolded. Both points of the calipers were applied until the first sign of blanching, and the patient was asked whether one/two points were felt; the minimum separation at which both points were felt was recorded [27, 28]. The same points on the contralateral side of the body were tested for use as a control. The average minimum separation was then calculated for both ipsilateral and contralateral sides, and the difference between the two values was used to calculate a two-point discrimination percentage deficit.

### 2.5. Data Collection and Statistics

The NHS Lothian TRAK system was utilized to obtain the following additional information: tumour volume, tumour grade, operative anaesthetic, and adjuvant radiotherapy.

Baseline characteristics of study patients were summarized with frequencies and percentages for categorical variables and as mean and standard deviation for continuous variables. Normality of data was checked using Shapiro-Wilk and Kolmogorov-Smirnov tests. The demographic and clinical characteristics of patients were evaluated using bivariate analysis. The significance of categorical variables was assessed using the Chi-squared tests or two-sided Fisher's exact tests (where less than five cases occurred in a cell). The significance of continuous variables was assessed using Student's *t*-test or Mann-Whitney *U* (MWU) test for continuous nonparametric data. Significance was set at *P* ≤ 0.05, and two-tailed *P* values were reported throughout.

## 3. Results

### 3.1. Objective Sensory Deficit

**Figure 1:**
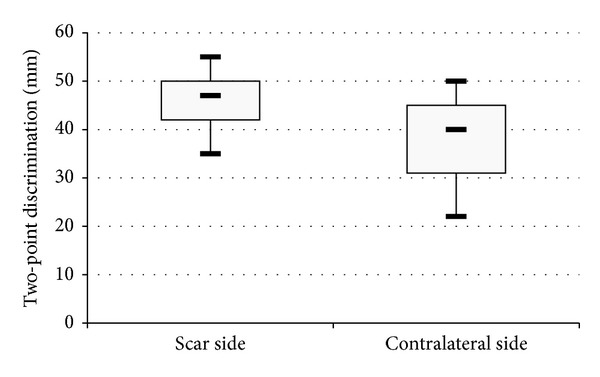
Box and Whisker plot showing values for two-point discrimination around the scar and on the contralateral side of the body. Discriminatory ability was markedly decreased in the area around the scar compared to the unaffected contralateral side of the body (*P* = 0.002). Scar side two-point discrimination: median (*Q*2) = 47 mm, lower quartile (*Q*1) = 42 mm, and upper quartile (*Q*3) = 50 mm. Contralateral side two-point discrimination: *Q*2 = 40 mm, *Q*1 = 31 mm, and *Q*3 = 45 mm.

86% of patients had some deficit in LT, PP, or both around the scar compared to the unaffected contralateral side of the body. 59% had deficit in LT sensation, with a mean area deficit of 12.99 cm^2^. 73% had deficit in PP sensation, with a mean area deficit of 14.28 cm^2^. 93% had a decreased discriminatory ability in the affected limb compared with the contralateral side of the body. Utilising a Wilcoxon's test, the difference in two-point discrimination between the scar side and contralateral side was shown to be statistically significant (*z* = −3.078, *P* = 0.002) (Figure 1).

**Figure 2:**
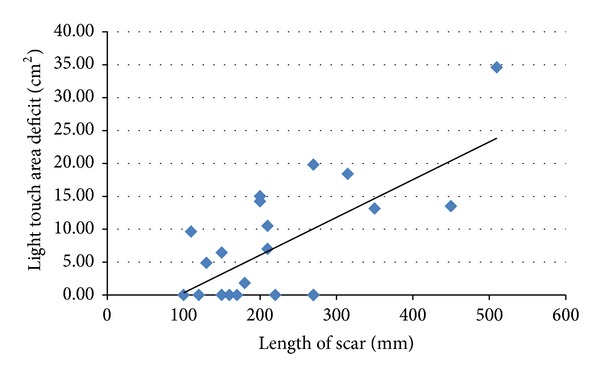
Scatter plot showing the relationship between scar length and light touch area deficit. There is a statistically significant positive correlation between the length of the scar and light touch area deficit. Spearman's correlation coefficient = 0.451. *P* = 0.035. As the length of the scar increased, the area of light touch deficit around the scar also increased.

**Figure 3:**
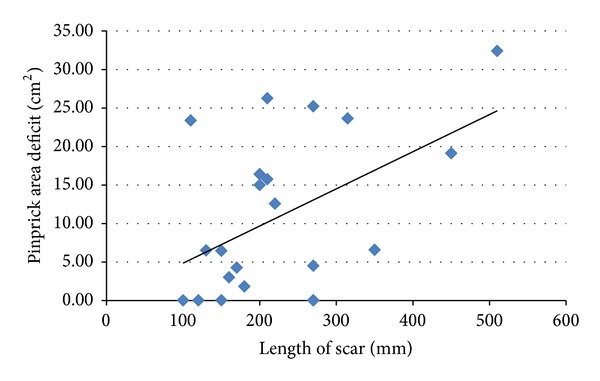
Scatter plot showing the relationship between scar length and pinprick area deficit. There is a statistically significant positive correlation between the length of the scar and pinprick area deficit. Spearman's correlation coefficient = 0.442.  *P* = 0.04. As the length of the scar increased, the area of pinprick deficit around the scar also increased.

A Spearman's test was performed to determine the relationship between scar length and objective sensory dysfunction. Both LT and PP deficits were significantly positively correlated with the size of the scar (*P* = 0.035 and *P* = 0.04, resp.) (Figures 2 and 3).

**Figure 4:**
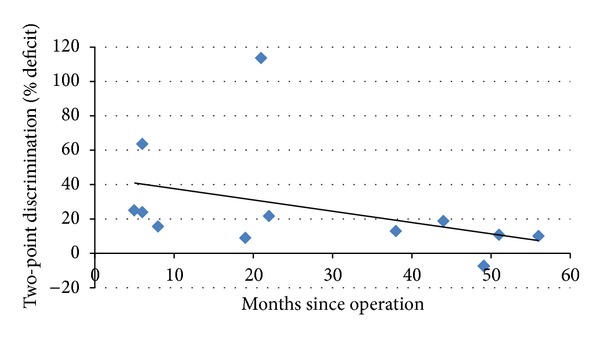
Scatter plot showing the relationship between months since operation and two-point discrimination percentage deficit. Correlation coefficient = −0.664.  *P* = 0.024.

We then investigated whether the objective sensory deficit showed any improvement over time. Spearman's test was performed to determine the relationship between months since operation and two-point discrimination percentage deficit; there was a negative correlation (−0.664), which was statistically significant (*P* = 0.024).  Figure 4 shows the relationship.

Two-point discrimination deficit decreases as time elapses after sarcoma surgery (i.e., the difference in two-point discrimination between the operated side and contralateral side of the body decreases over time after surgery).

### 3.2. Subjective Sensory Deficit

91% of patients had some subjective deficit around the scar compared with the contralateral side. 36% had sensation loss, 55% had numbness, and 59% had tingling. 59% felt pain around the scar (mean severity was 4.3/10). 59% felt their sensory symptoms had inconvenienced them and/or affected limb function.

**Figure 5:**
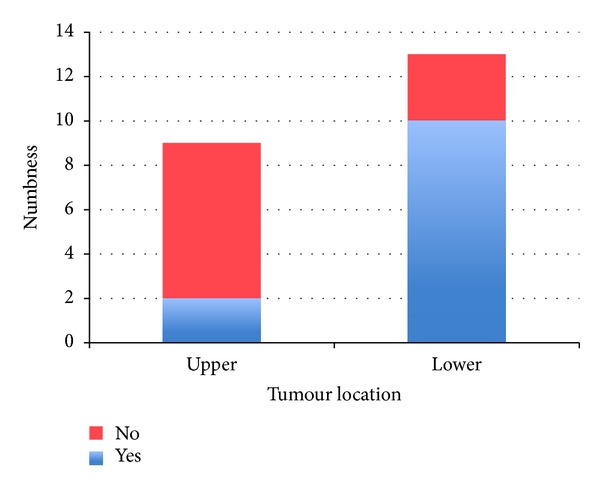
Stacked bar chart showing the relationship between tumour location and numbness. The subjective feeling of numbness was significantly more likely to occur in patients with lower body tumours than in patients with upper body tumours. *P* = 0.027.

There was a significant relationship between tumour location and numbness, as shown by Fisher's test (*P* = 0.027). 22% of upper body tumours occurred with numbness, whereas 77% of lower body tumours occurred with numbness. The relationship is shown in Figure 5.

Mean tumour volume also differed with location; upper body tumours had a mean volume of 470.5 cm^2^, whereas lower body tumours had a mean volume of 402.7 cm^2^. Tumour grade also differed with location; 100% of upper body tumours were high grade, whereas 73% of lower body tumours were high grade.

### 3.3. Do Subjective Deficits Relate to Objective Deficits?

We tested whether any aspect of a subjective deficit was significantly related to any aspect of objective deficit. There were no significant relationships between any subjective or objective sensation deficits.

## 4. Discussion

86% had some LT/PP deficit. Interestingly, the deficits in LT and PP were similar but not congruent. More patients had a PP deficit and the mean area of PP deficit was larger. This lack of congruence may arise as LT sensation and PP sensation are conveyed via different sensory pathways. Pacinian/Meissner corpuscles mediate LT [29], whereas PP is mediated by A-fibre nociceptors [30]. Our results suggest that the PP pathway is more likely to be damaged via sarcoma surgery. However another plausible explanation is that both LT and PP pathways are damaged to similar extent, but the LT pathway is more resilient; indeed LT receptors have been shown to function in an atrophied state for lengthy periods [29]. Identification of these resilient features of LT receptors may be valuable in the future for understanding and managing peripheral neuropathies.

Results show that 93% had significantly decreased discriminatory ability in the affected limb, indicating that sarcoma surgery disrupts tactile discrimination. This finding is corroborated by a study observing median nerve function after injury, which also reported a tactile discrimination loss [31].

Although two-point discrimination is an extremely popular/practical test, some studies highlight procedural flaws [32]. Studies show discriminatory ability diminishes with age [33]; however, the current study shows a nonsignificant relationship between patient age and discriminatory deficit. Patient concentration has also been shown to influence results [34]. Another study showed that test repetition could improve discriminatory ability [35]. This phenomenon may have affected our results, as the contralateral (unaffected) limb was always tested second, and hence the improved discrimination could be partially due to repetition. Future improved methodology would remove this confounding factor via randomising testing order.

Results show that as scar length increases LT/PP deficit increases. Results also show that patients with no inconvenience had a smaller mean scar length (183 mm) than patients with inconvenience (258 mm), although this was nonsignificant. Other studies have described an increased frequency of complications with larger scars such as increased healing times [36], psychological issues [37], and poorer cosmetic results [38]. It may therefore be worthwhile to surgically minimize scar length, without compromising tumour excision. Indeed other surgical forms are focusing on reducing scar length to improve outcome, for example, in haemangioma removal [39] and abdominoplasty [40].

Results also showed negative correlations between months since operation and two-point discrimination/LT/PP deficits; however only the relationship between two-point discrimination and months since operation was significant. An explanation is that there is a recovery of sensory function over time (tolerance to sensory dysfunction, although a recognized phenomenon [41], is not a plausible explanation, as sensation deficit was objectively measured). Recovery was also reported in a study on schwannomas, where 73.2% developed a new neurological deficit after enucleation, but at final follow-up 70% had no deficit [17]. Recovery of neurological function has also occurred after peripheral nerve injury [29, 42]. Another study, where sensory recovery occurred, emphasizes the role that injury mechanism, age, and sensory reeducation have [43]. The possibility of sensation recovery demands that sensory reeducation be a mandatory component of the management strategy for those with deficits after sarcoma surgery.

91% had some form of subjective sensory deficit; in another study 73.2% had a new subjective neurological deficit following tumour surgery [17]. In both studies the subjective sensory characteristic of tingling was most common. Results showed that numbness was significantly more likely in lower body than upper body tumours. An explanatory theory is that lower body tumours are larger and more often high grade at presentation (both characteristics are linked with more complications [44–47]). Our study could not confirm either of these points. Upper body tumours had a larger mean volume (however, volume data was only obtained for 2 upper body tumours); and 100% of upper body tumours were high grade, whereas only 73% of lower body tumours were high grade. A plausible explanation for our results is that, due to the greater density of sensory receptors in the upper body [48], a surgical excision would damage a smaller proportion of receptors (although actually a larger total number) in the upper body; therefore, less sensory dysfunction occurs.

Evaluating sensation deficit is fundamental in many neurological examinations, and numerous objective and subjective methods exist; but should objective/subjective modalities be utilised together to confer a more comprehensive insight into sensory function? Our results show that there were no significant relationships between subjective and objective deficits, indicating that these modalities cover different aspects of sensory dysfunction. There was, however, a smaller mean LT deficit in patients with no sensation loss (5.8 cm^2^) than in patients with sensation loss (10.89 cm^2^), indicating some overlap in assessment tools. These results validate findings by other researchers that although objective sensation tests are a useful tool to evaluate sensory dysfunction, they only partially replicate the complex perception of sensation [49], and hence they must be utilised in conjunction with subjective tools, in order to thoroughly understand a patient's sensory deficit.

## 5. Conclusion

We have demonstrated that sensory neuropathy is a relatively common complication of sarcoma excision surgery. Our results indicate that as scar size increases, sensation deficit also increases; therefore, efforts to minimise the length of scar during sarcoma excision surgery are important. Our results demonstrate that the sensory neuropathy improves as time after sarcoma surgery elapses; therefore, there could be an element of sensation recovery. It is therefore important that sensory reeducation techniques be considered as part of the postoperative management plans in these patients. Finally our results showed that there were no significant relationships between any objective and subjective deficits; therefore, when assessing sensation deficits after sarcoma surgery it would be pertinent to use both objective and subjective assessment techniques.

## References

[1] Arndt CAS, Crist WM (1999). Common musculoskeletal tumors of childhood and adolescence. *The New England Journal of Medicine*.

[2] Ferrari A, Bisogno G, Macaluso A (2007). Soft-tissue sarcomas in children and adolescents with neurofibromatosis type 1. *Cancer*.

[3] Almefty K, Pravdenkova S, Colli BO, Al-Mefty O, Gokden M (2007). Chordoma and chondrosarcoma: similar, but quite different, skull base tumors. *Cancer*.

[4] Carneiro A, Bendahl P-O, Engellau J (2011). A prognostic model for soft tissue sarcoma of the extremities and trunk wall based on size, vascular invasion, necrosis, and growth pattern. *Cancer*.

[5] Golledge J, Fisher C, Rhys-Evans PH (1995). Head and neck liposarcoma. *Cancer*.

[6] Lewis VO (2009). What’s new in musculoskeletal oncology. *Journal of Bone and Joint Surgery A*.

[7] Sarcoma UK What is sarcoma?. http://www.sarcoma.org.uk/what-is-sarcoma.

[8] Brockstein B (2004). Management of sarcomas of the head and neck. *Current Oncology Reports*.

[9] Jemal A, Siegel R, Xu J, Ward E (2010). Cancer statistics, 2010. *CA: Cancer Journal for Clinicians*.

[10] Bleyer A, Montello M, Budd T, Saxman S (2005). National survival trends of young adults with sarcoma: lack of progress is associated with lack of clinical trial participation. *Cancer*.

[11] http://cancerhelp.cancerresearchuk.org/type/sarcoma/treatment/statistics-and-outlook-for-soft-tissue-sarcoma#gen.

[12] Kasper B, Ho AD, Egerer G (2005). Is there an indication for high-dose chemotherapy in the treatment of bone and soft-tissue sarcoma?. *Oncology*.

[13] Grimer RJ (2005). Surgical options for children with osteosarcoma. *The Lancet Oncology*.

[14] Bielack SS, Kempf-Bielack B, Delling G (2002). Prognostic factors in high-grade osteosarcoma of the extremities or trunk: an analysis of 1,702 patients treated on neoadjuvant cooperative osteosarcoma study group protocols. *Journal of Clinical Oncology*.

[15] Wilkins RM, Cullen JW, Odom L (2003). Superior survival in treatment of primary nonmetastatic pediatric osteosarcoma of the extremity. *Annals of Surgical Oncology*.

[16] Dozor R (1990). The biopyschosocial model. *Journal of Family Practice*.

[17] Park MJ, Seo KN, Kang HJ (2009). Neurological deficit after surgical enucleation of schwannomas of the upper limb. *Journal of Bone and Joint Surgery B*.

[18] Sam RC, Silverman SH, Bradbury AW (2004). Nerve injuries and varicose vein surgery. *European Journal of Vascular and Endovascular Surgery*.

[19] Morrison C, Dalsing MC (2003). Signs and symptoms of saphenous nerve injury after greater saphenous vein stripping: prevalence, severity, and relevance for modern practice. *Journal of Vascular Surgery*.

[20] Moawad MR, Masannat YA, Alhamdani A, Gibbons CP (2008). Nerve injury in lower limb vascular surgery. *Surgeon*.

[21] Mendell JR, Sahenk Z (2003). Painful sensory neuropathy. *The New England Journal of Medicine*.

[22] Atrey A, Gupte CM, Corbett SA (2010). Review of successful litigation against english health trusts in the treatment of adults with orthopaedic pathology: clinical governance lessons learned. *Journal of Bone and Joint Surgery A*.

[23] Calfee RP, Manske PR, Gelberman RH, van Steyn MO, Steffen J, Goldfarb CA (2010). Clinical assessment of the ulnar nerve at the elbow: reliability of instability testing and the association of hypermobility with clinical symptoms. *Journal of Bone and Joint Surgery A*.

[24] Poort LJ, van Neck JW, van der Wal KGH (2009). Sensory testing of inferior alveolar nerve injuries: a review of methods used in prospective studies. *Journal of Oral and Maxillofacial Surgery*.

[25] Wade JB, Price DD, Hamer RM, Schwartz SM, Hart RP (1990). An emotional component analysis of chronic pain. *Pain*.

[26] Price DD, Bush FM, Long S, Harkins SW (1994). A comparison of pain measurement characteristics of mechanical visual analogue and simple numerical rating scales. *Pain*.

[27] Macleod J, Douglas G, Nicol F, Robertson C *Macleod's Clinical Examination*.

[28] Bell-Krotoski J, Weinstein S, Weinstein C (1993). Testing sensibility, including touch-pressure, two-point discrimination, point localization, and vibration. *Journal of Hand Therapy*.

[29] Burnett MG, Zager EL (2004). Pathophysiology of peripheral nerve injury: a brief review. *Neurosurg Focus*.

[30] Magerl W, Fuchs PN, Meyer RA, Treede R-D (2001). Roles of capsaicin-insensitive nociceptors in cutaneous pain and secondary hyperalgesia. *Brain*.

[31] Hansson T, Brismar T (2003). Loss of sensory discrimination after median nerve injury and activation in the primary somatosensory cortex on functional magnetic resonance imaging. *Journal of Neurosurgery*.

[32] Van Boven RW, Johnson KO (1994). The limit of tactile spatial resolution in humans: grating orientation discrimination at the lip, tongue, and finger. *Neurology*.

[33] Lundborg G, Rosén B (2004). The two-point discrimination test: time for a re-appraisal?. *Journal of Hand Surgery*.

[34] Birch R *Surgical Disorders of the Peripheral Nerves*.

[35] Craig JC, Johnson KO (2000). The two-point threshold: not a measure of tactile spatial resolution. *Current Directions in Psychological Science*.

[36] Kim Y-H, Sohn K-S, Kim J-S (2006). Short-term results of primary total knee Arthroplasties Performed with a Mini-Incision or a Standard Incision. *Journal of Arthroplasty*.

[37] Dorr LD, Thomas D, Long WT, Polatin PB, Sirianni LE (2007). Psychologic reasons for patients preferring minimally invasive total hip arthroplasty. *Clinical Orthopaedics and Related Research*.

[38] Tung VS, Buchberg B, Masoomi H (2011). No visible scar (NVIS) colectomy: A new approach to minimal access surgery to the colon. *Surgical Innovation*.

[39] Mulliken JB, Rogers GF, Marler JJ (2002). Circular excision of hemangioma and purse-string closure: the smallest possible scar. *Plastic and Reconstructive Surgery*.

[40] Momeni A, Torio-Padron N, Bannasch H, Borges J, Stark GB (2008). A new method for reducing postoperative complications and scar length in abdominoplasty. *Plastic and Reconstructive Surgery*.

[41] Woodrow KM, Friedman GD, Siegelaub AB, Collen MF (1972). Pain tolerance: differences according to age, sex and race. *Psychosomatic Medicine*.

[42] Robinson MD, Shannon S (2002). Rehabilitation of peripheral nerve injuries. *Physical Medicine and Rehabilitation Clinics of North America*.

[43] Dellon AL (1986). Sensory recovery in replanted digits and transplanted toes: a review. *Journal of reconstructive microsurgery*.

[44] Collin C, Godbold J, Hajdu S, Brennan M (1987). Localized extremity soft tissue sarcoma: an analysis of factors affecting survival. *Journal of Clinical Oncology*.

[45] Collin CF, Friedrich C, Godbold J, Hajdu S, Brennan MF (1988). Prognostic factors for local recurrence and survival in patients with localized extremity soft-tissue sarcoma. *Seminars in Surgical Oncology*.

[46] Geer RJ, Woodruff J, Casper ES, Brennan MF (1992). Management of small soft-tissue sarcoma of the extremity in adults. *Archives of Surgery*.

[47] Pezzi CM, Rawlings MS, Esgro JJ, Pollock RE, Romsdahl MM (1992). Prognostic factors in 227 patients with malignant fibrous histiocytoma. *Cancer*.

[48] Kenshalo D (1968). *The Skin Senses*.

[49] Curatolo M, Petersen-Felix S, Arendt-Nielsen L (2004). Assessment of regional analgesia in clinical practice and research. *British Medical Bulletin*.

